# Development and Validation of a Method of Liquid Chromatography Coupled with Tandem Mass Spectrometry for Quantification of ST-246 (Tecovirimat) in Human Plasma

**DOI:** 10.3390/molecules27113577

**Published:** 2022-06-02

**Authors:** Galina A. Oleinik, Vladimir V. Koval, Svetlana V. Usova, Larisa N. Shishkina, Alexander A. Chernonosov

**Affiliations:** 1 Institute of Chemical Biology and Fundamental Medicine SB RAS, Lavrentiev Ave. 8, Novosibirsk 630090, Russia; zakabluk@niboch.nsc.ru (G.A.O.); koval@niboch.nsc.ru (V.V.K.); 2 Novosibirsk State University, Pirogova Str. 2, Novosibirsk 630090, Russia; 3 State Research Center of Virology and Biotechnology VECTOR, Koltsovo 630559, Novosibirsk Oblast, Russia; usova_sv@vector.nsc.ru (S.V.U.); shish@vector.nsc.ru (L.N.S.)

**Keywords:** MRM, LC-MS/MS, tecovirimat, ST-246, NIOCH-14, plasma

## Abstract

The aim of this work was to develop and validate a sensitive and robust method of liquid chromatography coupled with tandem mass spectrometry to quantitate ST-246 (tecovirimat) in plasma using an internal standard (2-hydroxy-N-{3,5-dioxo-4-azatetracyclo [5.3.2.02.6.08.10]dodec-11-en-4-yl}-5-methylbenzamide). The method was validated in negative multiple reaction monitoring mode following recommendations of the European Medicines Agency for the validation of bioanalytical methods. The calibration curve for the analyte was linear in the 10–2500 ng/mL range with determination coefficient R^2^ > 0.99. Intra- and inter-day accuracy and precision for three concentrations of quality control were <15%. Testing of long-term stability of ST-246 (tecovirimat) in plasma showed no degradation at −20 °C for at least 3 months. The method was applied to a clinical assay of a new antipoxvirus compound, NIOCH-14. Thus, the proposed method is suitable for therapeutic drug monitoring of ST-246 (tecovirimat) itself and of NIOCH-14 as its metabolic precursor.

## 1. Introduction

Smallpox is a highly contagious viral infection caused by the *Orthopoxvirus* genus [1]. A strain of the variola virus led to classic smallpox and was fatal in 30% of cases. The virus is believed to have spread in the population between the 4th and 20th century. Progress in the fight against smallpox was made possible by Jenner’s invention of the vaccine in 1796 [2]. Nonetheless, only in 1980, did the World Health Organization (WHO) declare smallpox eradicated and recommended that the smallpox vaccination be stopped [3]. Although the WHO announced the eradication of smallpox in 1980, there are still two sites for the storage of the variola virus in the world: the State Research Center of Virology and Biotechnology VECTOR (Russia) and the Centers for Disease Control and Prevention (USA) [4]. The danger of the use of this virus as a biological weapon cannot be ruled out. Therefore, the development of new drugs against smallpox is required [5,6]. Lately, due to the cessation of the vaccination, the number of outbreaks of orthopoxvirus infections caused by existing pathogenic viruses has increased, as has the number of outbreaks caused by related zoonotic viruses that are also dangerous to humans [7]. Vaccination programs have provided protection against the virus for 3 to 5 years [8], and in case of infection, could be effective when administered within a few hours. The ACAM2000 vaccine, licensed for use in the USA in 2007, does not have great advantages over the freeze-dried Dryvax vaccine [9]. Thus, for a long time, there have been no safe and effective drugs against smallpox, resulting in active development of (and search for) new medicines. In the list of potentially active substances against smallpox, cidofovir and brincidofovir [10,11] have been found to be effective, but they are not yet approved for the treatment of orthopoxvirus infections. In addition, SIGA Technologies Netherlands B.V. has developed a new drug with activity against smallpox: tecovirimat (first code name ST-246). In July 2018, tecovirimat was approved by the FDA for the treatment of orthopoxvirus infections and thus became the first US drug against these viruses [12]. Tecovirimat suppresses the activity of the P37 protein, which is the main envelope protein of the orthopoxvirus. This action prevents the formation of virions capable of leaving the envelope, and this process is required for the replication of the virus in the host. The efficacy of tecovirimat against a wide range of orthopoxviruses has been demonstrated in several animal models, including a model involving the variola virus in nonhuman primates [13,14].

Another potential medication is a prodrug of tecovirimat with the code name NIOCH-14, which is reported to have a similar antipoxvirus activity [15]. This compound is fully metabolized into tecovirimat in the body, and therefore the pharmacokinetics of NIOCH-14 could be described by the quantification of tecovirimat as its main immediate metabolite [16].

During the development of ST-246 (tecovirimat), mass spectrometric methods of detection have been mentioned only in a few articles [17,18,19,20,21]. The ST-246 (tecovirimat) was detected in MRM mode by monitoring the *m/z* 375.1 → 282.9 [17,19] or *m/z* 375.0 → 283.2 [18,20,21] transition. Any additional transitions for qualification were not applied. The extraction of ST-246 (tecovirimat) was performed by the liquid–liquid extraction method [17,18,19,20,21] as well as by protein precipitation by means of a ninefold volume of methanol followed by the addition of a compensation solution (0.05% acetic acid in 0.05% ammonium hydroxide:methanol at 36:55, *v/v*) [18,20,21]. Nevertheless, in these articles, validation was not fully described, and for human plasma samples, the validated range was 50 to 4000 ng/mL [17,19,21]. Accordingly, a high limit of quantitation (LOQ) is not appropriate for most of clinical studies because very often tecovirimat concentration decreases below 50 ng/mL. Therefore, here we report a fully validated method of ST-246 (tecovirimat) quantification in human plasma with a lower LOQ, and this assay is applicable to research on NIOCH-14 as a prodrug of tecovirimat.

## 2. Results and Discussion

### 2.1. The Extraction Procedure

**Figure 1:**
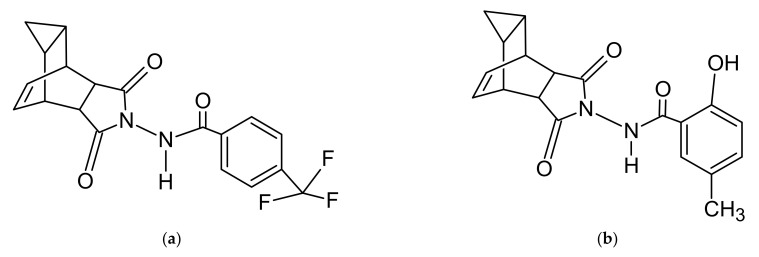
Chemical structure of ST-246 (tecovirimat) (**a**) and IS (**b**).

The most widely used technique for ST-246 (tecovirimat) extraction is a liquid–liquid extraction method [17,18,19,20,21]. In refs. [17,19], human plasma was subjected to extraction with an ammonium hydroxide solution in methanol and a solution containing 0.05% of ammonium hydroxide and 0.05% of acetic acid in methanol, respectively. By contrast, in other studies, the extraction of ST-246 (tecovirimat) from plasma was also carried out by protein precipitation by means of a ninefold volume of methanol followed by the addition of a compensation solution (0.05% acetic acid in 0.05% ammonium hydroxide:methanol at 36:55, *v/v*) [18,20,21]. Here we present an extraction by proteins precipitation method, consisting of mixing a human-plasma sample with an equivalent volume of methanol to obtain a fine suspension, followed by the addition of the extraction solution (acetonitrile containing an internal standard (IS), 2-hydroxy-N-{3,5-dioxo-4-azatetracyclo [5.3.2.02.6.08.10]dodec-11-en-4-yl}-5-methylbenzamide, Figure 1). The final dilution in the analyzed solution was sixfold relative to the plasma concentration of ST-246 (tecovirimat). At the step of sample extraction optimization, only extraction time showed a significant effect on tecovirimat recovery. Accordingly, optimal extraction time was found (60 min) and along with other parameters (solvent and volume of solvent) yielded the simplest extraction technique with good recovery.

### 2.2. Liquid Chromatography and Mass Spectrometry Conditions

To optimize chromatography parameters, we tested several gradients, and as a result, a simple linear gradient from 2% to 100% of acetonitrile during 7 min afforded adequate separation of ST-246 (tecovirimat) and the IS, which have very similar structures. Total chromatography time was 10 min, where the column was re-equilibrated during the last 3 min.

**Figure 2:**
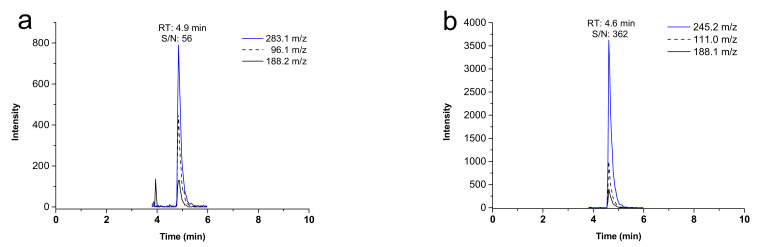
Representative chromatograms of (**a**) transitions *m/z* 375.1 → 283.1, 375.1 → 188.2, and 375.1 → 96.1 for the LOQ at 10 ng/mL ST-246 (tecovirimat) and (**b**) transitions *m/z* 337.2 → 245.2, 337.2 → 188.1, and 337.2 → 111.0 of 30 ng/mL IS with the corresponding retention time (RT) and signal-to-noise (S/N) ratio.

The mass spectrometric analysis was carried out in electrospray negative ion mode. Multiple reaction monitoring (MRM) mode was chosen for ST-246 (tecovirimat) quantification and structure confirmation. Transition *m/z* 375.1 → 283.1, which generated a more intense signal, was employed for ST-246 (tecovirimat) quantification, whereas transitions *m/z* 375.1 → 188.2 and 375.1 → 96.1 were used for the structure confirmation (Figure 2a). The *m/z* 337.2 → 245.2 transition was selected for IS quantification, whereas transitions *m/z* 337.2 → 188.1 and 337.2 → 111.0 for the confirmation of IS structure (Figure 2b).

### 2.3. Linearity and Limits of Quantitative Detection

The LOQ had to meet the following criteria: the signal-to-noise (S/N) ratio above 10 and measurement error not exceeding 20%. Therefore, the lowest concentration of the linear concentration range that satisfied these requirements was assumed to be the LOQ. The S/N ratio was very high for all tested concentrations, but the tolerance of accuracy and precision for concentration below 10 ng/mL exceeded 20%; thus, 10 ng/mL was chosen as the LOQ. Calibration standards did not exceed the limits established by the European Medicines Agency (EMA), either for accuracy or for precision within the 10–2500 ng/mL range with weighting coefficient 1/x and with determination coefficient R^2^ > 0.99. The calibration standards above 2500 ng/mL no longer corresponded to a linear range; therefore, the linear range was 10–2500 ng/mL. In some studies [17,19,21], a validated linear range for ST-246 was 50 to 4000 ng/mL, whereas the application of our assay to real-world samples indicated that the maximal ST-246 concentration did not exceed 1000 ng/mL. Consequently, the linear range 10–2500 ng/mL is more suitable for clinical application.

### 2.4. Accuracy and Precision

Intra-day variability was assessed by analyzing low quality control (LQC) concentration, medium quality control (MQC) concentration, high quality control (HQC) concentration, and the LOQ as six artificial replicates (artificial samples prepared and analyzed in an identical way in parallel; hereafter: replicates) in the same analytical run. Accuracy and precision for ST-246 (tecovirimat) at all quality control concentrations were within 14.5% and 7.8%, respectively. At the LOQ, accuracy and precision did not exceed 19.9% and 13.0%, respectively. Inter-day variability was evaluated in analytical runs conducted on three consecutive days at the same concentrations that were used for the intra-day variability estimation. Accuracy and precision at all quality control concentrations were within 6.6% and 12.0%, respectively. At the LOQ, accuracy and precision were <19.0% and <7.1%, respectively. The complete results on inter-day and intra-day variability estimates are displayed in Table 1 (units are explained in Section 3).

### 2.5. Recovery and the Matrix Effect

These parameters were evaluated at LQC, MQC, HQC, and LOQ levels. The matrix effect was estimated by a comparison of the peak area of the ST-246/IS ratio (when ST-246 (tecovirimat) was added into a blank plasma extract) with that obtained by direct injection of chemical standards into the pure solvent. The ST-246 (tecovirimat) recovery was evaluated by comparing the peak area of the ST-246/IS ratio in samples that underwent standard extraction with that of samples in which ST-246 (tecovirimat) was added to the blank plasma extract. As presented in Table 2, the matrix effect was negligible, ranging between 95.6% and 104.3%, and the recovery ranged between 82.2% and 104.5%. The recovery decreased with increasing ST-246 (tecovirimat) concentration but did not fall below 82%. Unfortunately, we could not compare the recovery rates and matrix effect with other studies [18,19,21] because their authors did not specify these parameters.

### 2.6. Stability

Stability experiments included freeze–thaw cycles, short-term incubation in an autosampler at 4 °C, and long-term storage at −20 °C. Plasma samples containing ST-246 (tecovirimat) were stable at all quality control levels and LOQ after three consecutive freeze-thaw cycles showing 96–115% of nominal concentrations. In stability testing in the autosampler, ST-246 (tecovirimat) manifested good stability during 48 h (Table 3). At −20 °C in the refrigerator ST-246 (tecovirimat) was stable at least for 90 days (Table 4).

### 2.7. Application of the Method

**Figure 3:**
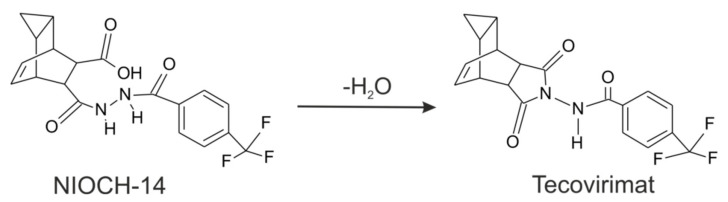
Metabolic conversion of NIOCH-14 into ST-246 (tecovirimat).

Because NIOCH-14 is quickly and fully metabolized into tecovirimat in the body (Figure 3), ST-246 (tecovirimat) could serve as the main immediate metabolite for characterizing pharmacokinetic parameters of NIOCH-14 [15]. Following interaction of NIOCH-14 with blood plasma, its mass cannot be registered, so the only way to determine the pharmacokinetic parameters of NIOCH-14 is analyzing the change in the concentration of ST-246 (tecovirimat). On the other hand, the presented method for tecovirimat detection could be tested on samples obtained after oral administration of NIOCH-14 substance.

Accordingly, the validated method was applied to plasma samples obtained from four healthy volunteers during a clinical study of NIOCH-14 (see Section 3.2 below). The real-world samples were collected at various time points after single oral administration of 600 mg of NIOCH-14.

**Figure 4:**
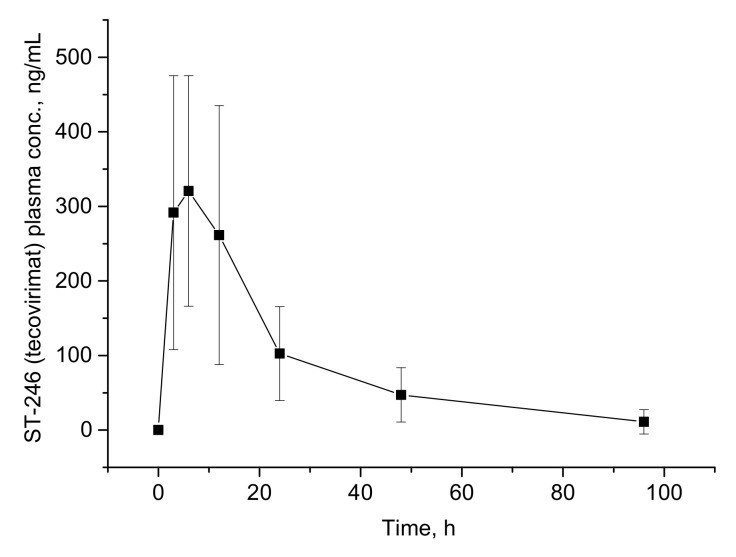
The mean plasma concentration–versus–time plot of ST-246 (tecovirimat) as the main immediate metabolite of NIOCH-14 after single oral administration of 600 mg of NIOCH-14 to healthy human volunteers (*n* = 4).

The mean ± SD of plasma concentrations (versus time) of the tecovirimat as the main immediate metabolite of NIOCH-14 are shown in Figure 4, which indicates the suitability of the newly developed method for pharmacokinetic studies of NIOCH-14 and tecovirimat itself in humans.

The calculated pharmacokinetic parameters are presented in Table 5. To compare them with pharmacokinetic parameters of ST-246 (tecovirimat) [17], we should assume that NIOCH-14 is fully converted into tecovirimat, consistently with ref. [16]. In this case, parameters of NIOCH-14 calculated from tecovirimat concentration were found here and are characterized by lower maximum concentration in plasma but longer half-life. According to the area under the curve, a dose of 600 mg of NIOCH-14 is approximately equivalent to 250 mg of ST-246. Of course, we do not know the conversion rate of NIOCH-14 to tecovirimat and the specific pathway of its metabolism in the body, which could affect the pharmacokinetic parameters.

## 3. Materials and Methods

### 3.1. Reagents

Acetonitrile for liquid-chromatography coupled with tandem mass spectrometry (LC-MS/MS) was purchased from Biosolve (Dieuze, France), HPLC grade methanol from J. T. Baker (Gliwice, Poland), and formic acid from Sigma–Aldrich (St. Louis, MO, USA). Water for the experiments was obtained using a Milli-Q purification system from Millipore Corp. (Bedford, MA, USA). Gaseous nitrogen (ultrapure, >99.9%) was produced by means of an Agilent 5183–2003 nitrogen generator (Agilent Technologies, Santa Clara, CA, USA), ST-246 (tecovirimat) and 2-hydroxy-N-{3,5-dioxo-4-azatetracyclo [5.3.2.02.6.08.10]dodec-11-en-4-yl}-5-methylbenzamide (used as an IS; Figure 3) were synthetized for research proposes by A.Ya. Tikhonov and B.A. Selivanov (N.N. Vorozhtsov Novosibirsk Institute of Organic Chemistry SB RAS) according to a previously described technique [22].

### 3.2. Plasma Samples

For method validation purposes, blank samples were obtained from healthy adult volunteers who were not being treated with either NIOCH-14 or tecovirimat. To test the assay on real samples, the plasma samples were obtained from four volunteers who were among the subjects recruited for the clinical study of NIOCH-14. The volunteers consisted of people aged 18 years or older with the following inclusion criteria: had no contraindications, were healthy, and had not taken other medications within the last 7 days. The study was conducted in accordance with Permission No. 357 (dated 22 July 2020) of the Ministry of Health of the Russian Federation and an Excerpt from Protocol No. 237 (dated 21 July 2020) of the Meeting of the Ethics Council of the Ministry of Health of the Russian Federation, which approved the conduct of clinical trials under Protocol No. NIOCH-01/20 “An open, simple, randomized study of the safety, tolerability, pharmacokinetics of NIOCH-14 in volunteers aged 18–50 years in parallel groups.” Plasma samples were collected at 0, 3, 6, 12, 24, 48, and 96 h after oral administration of 600 mg of NIOCH-14 and stored at −20 °C before analysis.

### 3.3. Preparation of Solutions

#### 3.3.1. Preparation of Stock Solutions

Stock solutions of ST-246 (tecovirimat) and IS at the concentration of 10 mg/mL were prepared by weighing 10.1, 10.1, 10.2, 10.1, and 10.3 mg quantities for analysis, followed by dissolving in acetonitrile. All stock solutions were stored at −20 °C.

#### 3.3.2. Preparation of Working Solutions

To prepare ST-246 (tecovirimat) working solutions, on the day of the assay, the stock solutions were diluted to 100 μg/mL with acetonitrile, followed by dilution to required concentrations. The IS working solution was prepared from the stock solution by dilution with acetonitrile to 30 ng/mL. The working solutions were stored at 4 °C until use.

#### 3.3.3. Preparation of Calibration Solutions

Solutions of ST-246 (tecovirimat) in acetonitrile for calibration were prepared on the day of analysis by dilution of each of the five stock solutions to 25,000, 10,000, 5000, 2500, 1000, 500, 200, and 100 ng/mL. Calibration standards’ solutions were obtained by tenfold dilution of the ST-246 (tecovirimat) acetonitrile solutions with plasma to obtain final plasma concentrations of 2500, 1000, 500, 250, 100, 50, 20, and 10 ng/mL. The tenfold dilution in plasma was performed by the addition of 5 μL of ST-246 (tecovirimat) solutions to 45 μL of plasma and shaking in a TS-100C thermo-shaker (BioSan, Latvia) for 30 min at 37 °C and 900 rpm. Calibration standards were prepared immediately prior to use.

#### 3.3.4. Preparation of Solutions for Validation

These solutions were prepared in the same way as calibration solutions were, by dilution of the ST-246 (tecovirimat) stock solution with acetonitrile to 20,000, 8000, 500, and 100 ng/mL, followed by tenfold dilution with plasma to obtain final plasma concentrations of 2000, 800, 50, and 10 ng/mL.

### 3.4. The Extraction Procedure

Methanol (50 μL) was added to 50 μL of plasma, and the solution was shaken to obtain a fine suspension. Then, 200 µL of the IS working solution was added for extraction by shaking at 40 °C and 900 rpm for 60 min. After centrifugation at 13,000× *g* for 5 min, an aliquot (130 μL) of the supernatant was transferred into vials and analyzed in an LC-MS/MS system.

### 3.5. LC-MS/MS in MRM Mode

The mass spectrometric analysis was carried out at the Basic Center for Mass Spectrometric Analysis (ICBFM SB RAS). Analyzes were performed by means of an Agilent 1200 HPLC instrument (Agilent Technologies, USA). A ProntoSil-120-3-C18 (2 × 75 mm, 3 μm) analytical column (EcoNova, Russia) with an Eclipse XDB-C18 guard column (Agilent Technologies, USA) was used for chromatographic separation. The mobile phase consisted of water (eluent A) and acetonitrile (eluent B). The eluent started from 2% B and the B concentration was increased linearly up to 100% from minute 0.3 to minute 7; at 7.01 min, it was returned to 2% B and stayed at that level until 10 min for column reconstitution. The flow rate was 300 μL/min throughout the analysis. The autosampler temperature was maintained at 6 °C. The column temperature was set to 50 °C. During the procedure, the signals were recorded from minute 4.2 to minute 5.8, i.e., within retention times of ST-246 and IS; the rest of the time, the eluent was poured into the drain. The total analysis time was 10 min.

The ST-246 (tecovirimat) was assayed by LC-MS/MS on an Agilent-1200 liquid chromatograph equipped with an Agilent 6410 QQQ mass spectrometer (Agilent Technologies, USA). The procedure was performed in MRM mode by electrospray ionization (ESI) in negative mode.

The analysis parameters were set as follows: the volume of the aliquot, 10 μL; capillary voltage, 4 kV. Nitrogen was supplied as a spray and carrier gas under a pressure of 45 psi at a flow rate of 6 L/min at 300 °C. The dwell time was set to 200 ms.

The measurements were carried out in the MRM mode for precursor ions with *m/z* 375.1 (ST-246) and 337.2 (IS) and the corresponding fragment. The transitions with the strongest signal were selected and used for quantitative confirmation of a compound: *m/z* 375.1 → 283.1 (ST-246, collision energy 20 eV, fragmentation voltage 135 V) and *m/z* 337.2 → 245.2 (IS, collision energy 20 eV, fragmentation voltage 135 V). The transitions *m/z* 375.1 → 188.2 and 375.1 → 96.1 as well as *m/z* 337.2 → 188.1 and 337.2 → 111.0 served as qualifiers for ST-246 (tecovirimat) and the IS, respectively.

### 3.6. Validation

To this end, linearity, accuracy, precision, recovery, the matrix effect, and sample stability were determined. During the development of this assay, the validation was carried out according to the EMA guidelines [23].

#### 3.6.1. Linearity and Limits of Quantitative Detection

The linearity of the newly developed method was assessed by plotting a calibration curve. The latter was constructed using eight calibration levels of the following concentrations: 2500, 1000, 500, 250, 100, 50, 20, and 10 ng/mL.

#### 3.6.2. Accuracy and Precision

To evaluate intra-day precision, the three levels of quality control (low, medium, and high) and a lower LOQ (50, 800, 2000, and 10 ng/mL, respectively) were analyzed in six replicates within 1 day.

The ratio (%) of the calculated mean concentration to the nominal concentration was defined as accuracy (% bias).

Inter-day accuracy was assessed by the assay of samples at the same concentrations in six replicates for three consecutive days. The concentrations were chosen following the EMA Guidelines for Validation of Bioanalytical Methods [23]. Accuracy was expressed as a percentage of nominal concentration (% bias); precision was calculated as percentage relative standard deviation (% RSD). Tolerances for both parameters were deemed acceptable within ±15%, except for the LOQ, for which they had to be within ±20%.

#### 3.6.3. Recovery and the Matrix Effect

To evaluate ST-246 (tecovirimat) recovery, we compared analyte signals’ area between samples where ST-246 (tecovirimat) was extracted from plasma and samples where ST-246 (tecovirimat) was added to blank plasma extracts. Recovery experiments were performed on five replicates in an assay of four concentrations (LOQ, LQC, MQC, and HQC).

The matrix effect was defined as a change in an analyte response owing to interfering components in the sample solution. For this purpose, we compared the response to ST-246 (tecovirimat) added to the blank plasma extract and the response to ST-246 (tecovirimat) added to the pure solution.

#### 3.6.4. Stability

The stability of ST-246 (tecovirimat) in plasma was assessed in each of the following ways: after three freeze–thaw cycles, after incubation at −4 °C for 48 h, or after storage at −20 °C for 1.5 months. Samples with quality control and LOQ concentrations were analyzed as four replicates.

## 4. Conclusions

In summary, we developed a simple and robust quantitative assay of ST-246 (tecovirimat) in human plasma with a one-step extraction procedure. This method was fully tested and described for the first time. This method was validated and manifested ≤15% accuracy and precision in the 10–2500 ng/mL concentration range; this performance is suitable for clinical, preclinical, pharmacokinetic, and toxicity studies on ST-246 (tecovirimat) and its prodrug NIOCH-14. The ST-246 (tecovirimat) was found to be stable for at least 90 days at −20 °С and for 48 h in an autosampler at 4 °C. The validated assay was successfully applied for therapeutic drug monitoring for the first time of the new antiviral drug NIOCH-14 in a clinical study and show the possibility of NIOCH-14 analyzing by detection of tecovirimat as its main metabolite.

## Figures and Tables

**Table 1:** Intra-day and inter-day accuracy and precision.

Concentration, ng/mL	Intra-Day						Inter-Day	
	1st Day		2nd Day		3rd Day			
	% bias	% RSD	% bias	% RSD	% bias	% RSD	% bias	% RSD
10	−18.4	3.4	−17.6	13.0	−19.9	5.6	−19.0	7.1
50	14.0	7.8	−12.5	2.5	−0.1	5.2	0.3	11.0
800	13.4	4.0	−14.5	2.6	−4.7	4.3	−2.6	10.9
2000	12.6	1.6	−14.5	4.0	−12.8	3.5	−6.6	12.0

**Table 2:** Recovery and the matrix effect.

Concentration, ng/mL	Recovery (%)	Matrix Effect (%)
10	104.5	96.9
50	87.5	104.3
800	82.8	104.1
2000	82.2	95.6

**Table 3:** Stability of ST-246 (tecovirimat) in plasma at 4 °C for 48 h.

Concentration, ng/mL	0 h		5 h		24 h		48 h	
	(%)	RSD (%)	(%)	RSD (%)	(%)	RSD (%)	(%)	RSD (%)
10	−19.3	8.1	−19.8	0.9	−19.1	1.4	3.2	17.0
50	2.7	5.7	−2.9	3.0	4.3	8.4	12.8	13.7
800	−5.0	5.9	−4.4	2.5	−3.3	3.4	−2.7	5.8
2000	−12.0	4.9	−13.6	1.1	−13.2	2.3	−14.4	4.3

**Table 4:** Stability of ST-246 (tecovirimat) in plasma at −20 °C for three months.

Concentration, ng/mL	0 Day		14 Days		30 Days		45 Days		75 Days		90 Days	
	% bias	% RSD	% bias	% RSD	% bias	% RSD	% bias	% RSD	% bias	% RSD	% bias	% RSD
10	−20.0	1.4	−11.5	15.6	17.8	10.2	16.4	5.4	−12.6	19.9	13.3	11.3
50	2.3	0.8	7.5	11.6	7.1	10.2	13.0	3.6	3	5.9	12.5	3.9
800	−3.3	3.0	7.0	6.1	−2.1	11.5	8.3	3.9	2.6	3.3	11.6	1.1
2000	−13.8	1.0	1.2	6.1	−11.9	5.2	−2.2	4.8	−5.1	3.6	5.4	2.4

**Table 5:** Pharmacokinetic parameters of NIOCH-14, as calculated from data on its main immediate metabolite, tecovirimat.

Parameter	Value
Lambda_z, 1/h	0.05 ± 0.03
T_½_, h	17.2 ± 8.3
T_max_, h	5.25 ± 4.5
C_max_, ng/mL	415 ± 80
AUC_o-inf_, ng/(mL·h)	8864 ± 4117
MRT 0-inf, h	25 ± 12
V_z_/F, L	1798 ± 804
Cl/F, L/h	80 ± 37

## Data Availability

Data are available on request, owing to privacy and ethical restrictions.

## Sample Availability

Samples of the compounds are not available from the authors.
